# Detection and Yield of Colorectal Cancer Surveillance in Adults with *PTEN* Hamartoma Tumour Syndrome

**DOI:** 10.3390/cancers14164005

**Published:** 2022-08-19

**Authors:** Meggie M. C. M. Drissen, Janet R. Vos, Dorien T. J. van der Biessen-van Beek, Rachel S. van der Post, Iris D. Nagtegaal, Mariëtte C. A. van Kouwen, Tanya M. Bisseling, Nicoline Hoogerbrugge

**Affiliations:** 1 Department of Human Genetics, Radboud University Medical Center, 6500 Nijmegen, The Netherlands; 2 Radboud University Medical Center, Radboud Institute for Health Sciences, 6525 Nijmegen, The Netherlands; 3 Department of Gastroenterology and Hepatology, Radboud University Medical Center, 6500 Nijmegen, The Netherlands; 4 Department of Pathology, Radboud University Medical Center, 6500 Nijmegen, The Netherlands; 5 Radboud University Medical Center, Radboud Institute for Molecular Life Sciences, 6500 Nijmegen, The Netherlands

**Keywords:** PTEN phosphohydrolase, hamartoma syndrome, multiple, neoplastic syndromes, hereditary, colorectal neoplasms, colonic polyps, adenomatous polyps, population surveillance, colonoscopy, early detection of cancer

## Abstract

**Simple Summary:**

Colorectal cancer surveillance (CCS) with colonoscopy every five years is advised for *PTEN* Hamartoma Tumour Syndrome patients aged ≥40. However, data to support CCS guidelines are scarce and available colorectal cancer (CRC) risks are likely overestimated and low up to age 50. We aimed to assess the detection and yield of CCS for PHTS patients aged ≥40 seen at a PHTS expertise centre. Thirty-seven patients (median age 47 years) underwent 61 colonoscopies during 67 follow-up years. CCS yielded no CRCs. Adenomas were found in one-third of the cohort, including one advanced adenoma. The adenoma yield at baseline was similar to follow-up and higher above age 50 compared to age 50 or below. The low yield allows for a more personalised surveillance program. Combining our data with literature findings on CRC risk and progression, we suggest starting CCS at age 40 with variable follow-up intervals between 1 and 10 years depending on previous colonoscopy findings.

**Abstract:**

Colorectal cancer surveillance (CCS) with colonoscopy every five years is advised for *PTEN* Hamartoma Tumour Syndrome (PHTS) patients aged ≥40 due to an increased colorectal cancer (CRC) risk. However, data to support CCS guidelines are scarce and available CRC risks are low (0–5% at age 50) and likely overestimated. We aimed to assess the detection and yield of CCS for PHTS patients without a CRC history. A retrospective cohort study including PHTS patients aged ≥40 with CCS at a PHTS expertise centre between 2011 and 2022. Adenomas with a ≥10 mm size, (tubulo)villous histology, or high-grade dysplasia were considered advanced. During 67 follow-up years, 37 patients (median age 47 years) underwent 61 colonoscopies. CCS yielded no CRCs. Adenomas were diagnosed in 13/37 (35%) patients during 23/100 colonoscopies (95% CI: 14–36), including one advanced adenoma. Baseline adenoma detection rates were similar to follow-up and higher in patients aged above 50 (50/100, 95% CI: 24–76) vs. age 50 or below (11/100, 95% CI: 3–30; *p* = 0.021). The low CRC and advanced adenoma yield allow for a more personalised surveillance program. Following our findings combined with literature on CRC risk and progression, we suggest starting CCS at age 40 with variable follow-up intervals between 1 and 10 years depending on previous colonoscopy findings.

## 1. Introduction

*PTEN* Hamartoma Tumour Syndrome (PHTS) is a rare cancer predisposition syndrome with an estimated prevalence of 1 in 200,000 [1]. It is caused by pathogenic germline variants in the *PTEN* gene, a major tumour suppressor gene and regulator of cell proliferation and apoptosis through the PI3K/Akt pathway. Besides an increased risk of thyroid, breast, and endometrial cancer, PHTS patients are at increased risk of colorectal (CRC) cancer with estimates ranging between 9% and 32% at age 70, compared with 2% in the general population [2]. While in the general population CRC is diagnosed at a median age of 73 years, the median age in PHTS patients is lower, ranging between 46 to 58 years [2].

Furthermore, PHTS patients often develop benign lesions including adenomas and hamartomas (i.e., non-cancerous tissue overgrowth), such as thyroid nodules, colorectal hamartomas, and various skin lesions [3,4]. Colorectal adenomas and hamartomas have previously been found in 24–42% and 15–31% of PHTS cohorts, respectively [4,5,6,7]. Advanced colorectal adenomas, commonly defined as adenomas with either a ≥10 mm size, a villous or tubulovillous histology, or high-grade dysplasia, are considered direct precursor lesions of CRC [8,9,10,11]. In contrast, colorectal hamartomas might have no malignant potential [12].

Currently, PHTS patients are advised colorectal cancer surveillance (CCS) by means of regular colonoscopies to enable early CRC detection as well as CRC prevention by removal of precursor lesions, but the starting age and frequency varies among guidelines. The Dutch PHTS guideline (2015) advises performing a colonoscopy every five years in PHTS patients from age 40 [13]. Likewise, the National Comprehensive Cancer Network (NCCN) guideline (2022) and the US Multi-Society Task Force on CRC guideline (2022) advise performing colonoscopy surveillance at 5-year intervals, or more frequently depending on polyp burden, starting at age 35 [14,15]. The PHTS surveillance guideline (2020) of the European Reference Network for Genetic Tumour Risk Syndromes (ERN GENTURIS) suggests offering a baseline colonoscopy in patients aged 35–40 with follow-up as indicated by the gastroenterologist [16]. The United Kingdom Cancer Genetics Group (2017) advises performing a colonoscopy at age 35 and 55 and initiate polyp follow-up as required [17].

CCS guidelines for PHTS patients are mainly expert opinion-based since data on patients undergoing CCS are scarce, due to the rarity of PHTS. Furthermore, available CRC risks in PHTS patients indicate a CRC risk as low as 0–5% up to age 50, and are likely overestimated due to uncorrected ascertainment bias (i.e., inclusion of a selected cohort strongly enriched for cancer that may not be representative for the entire PHTS population) [2,16,18]. This emphasises the need for evaluation of CCS using observational data. We aimed to assess the detection and yield of CCS for adult PHTS patients without a history of CRC. More specifically, we aimed to assess the presence of colorectal lesions, in particular the presence of (advanced) adenomas and CRC.

## 2. Methods

### 2.1. Setting

This single-institution retrospective cohort study was performed in line with the national regulations on conducting scientific research. Adult PHTS patients aged ≥40 with a proven pathogenic *PTEN* variant who did not object against the use of their medical record data and received CCS at the Radboud university medical centre, a PHTS expertise centre, were included. Patients with a history of CRC prior to the start of CCS were excluded as they often receive a different CCS follow-up compared with patients without a history of CRC. Moreover, the risk of developing a second primary CRC might be higher compared with the risk of a first CRC in patients without a CRC history, as also shown for non-PHTS populations [19,20]. *PTEN* variant classification was performed using (previous versions of) the Association for Clinical Genetic Science/Dutch Society of Clinical Genetic Laboratory Specialists (ACGS/VKGL) guideline as the main directive [21]. Following the Dutch PHTS guideline [13], CCS by means of colonoscopy was offered to PHTS patients every five years or more frequently when indicated by the gastroenterologist or in case of complaints. Outcomes of surveillance colonoscopies performed between February 2012 and February 2022 were included. This included scheduled surveillance colonoscopies as well as colonoscopies in patients with colorectal complaints that also served the purpose of surveillance. Ethical approval for this study was obtained from the local Medical Ethics Review Committee (2022-15803). The need for informed consent was waived.

### 2.2. Data Collection and Outcomes

Data on clinical history and colonoscopy findings during CCS were collected from patients’ medical files. Colonoscopy findings concerned the presence and location of pathology-confirmed CRCs, adenomas, hamartomas (further classified as hamartomas of no special type (NST), ganglioneuromas, juvenile polyps, and lymphoid polyps), inflammatory polyps, hyperplastic polyps, and sessile serrated lesions. For colorectal adenomas and serrated lesions, additional information was collected including the histological subtype, degree of dysplasia, size (largest size in case >1 lesion was found), and number at each colonoscopy. Pathology diagnoses were not reassessed for the purpose of this study.

Colonoscopies were considered incomplete in case of insufficient bowel cleansing (i.e., a Boston Bowel Preparation score (BBPS) below 6 or, if the BBPS was not assessed, specified as such in the colonoscopy report) or if caecum/terminal ileum intubation was not reached [22,23]. Incomplete colonoscopies were excluded if this resulted in performing an additional colonoscopy within two years. Location of a colorectal lesion was categorised into proximal colon (i.e., proximal to the splenic flexure; caecum, ascending and transverse colon, and splenic flexure) and distal colon (i.e., distal to the splenic flexure; descending colon, sigmoid, and rectum). The time to adenoma detection was defined as the time between two subsequent colonoscopies where the former colonoscopy revealed no adenomas or all adenomas had been removed. Advanced adenomas were defined as adenomas with at least one of the following features: a ≥10 mm size, a villous or tubulovillous histology, or high-grade dysplasia [8,9,10,11].

### 2.3. Statistical Analyses

Continuous data are reported as median (interquartile range (IQR)) and categorical variables as counts and percentages. Detection rates of colorectal lesions are presented as the number of colonoscopies during which at least one lesion was detected per 100 colonoscopies. The detection and yield of CCS was assessed with and without stratification for gender. All statistical analyses were performed in R version 3.6.2 and two-sided *p*-values below 0.05 were considered statistically significant [24].

## 3. Results

### 3.1. Study Participants

**Figure 1:**
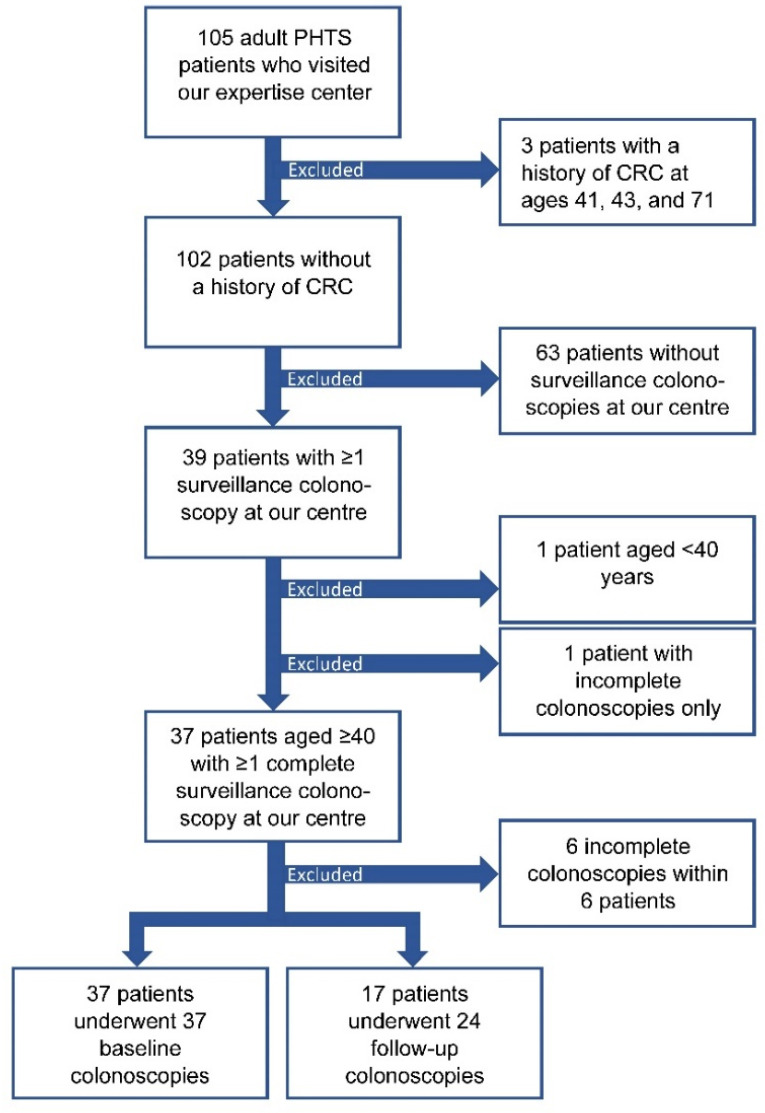
Flowchart of adult PHTS patients and colonoscopies included in this study. Abbreviations: PHTS: *PTEN* Hamartoma Tumour Syndrome; CRC: colorectal cancer. Colonoscopies were considered incomplete in case of insufficient bowel cleansing or if caecum/terminal ileum intubation was not reached, and a new colonoscopy had been scheduled within two years.

**Figure 2:**
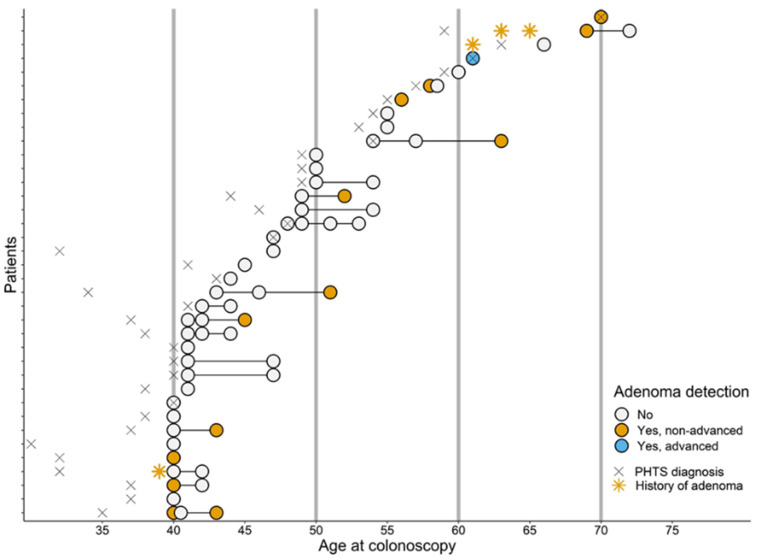
Timeline of colorectal cancer surveillance in adult PHTS patients and the detection of colorectal adenomas. Abbreviations: PHTS: *PTEN* Hamartoma Tumour Syndrome. Each horizontal line represents one unique patient and each dot represents one surveillance colonoscopy.

In total, 105 adult PHTS patients visited our expertise center, of whom three were excluded because of a history of CRC at ages 41, 43, and 71 (Figure 1). Of these 102 patients, 65 were excluded as they did not (yet) start CCS at our centre (N = 63) or started CCS at our centre but were aged below 40 years (N = 1) or only had incomplete colonoscopies available (N = 1). Of the remaining 37 patients, 24 (65%) were female (Table 1). Fifteen (41%) patients were index patients, i.e., the first patient in a family diagnosed with PHTS because of clinical signs, and 22 (59%) were non-index patients who underwent *PTEN* testing because of a familial mutation. The median age at PHTS diagnosis was 41 (IQR: 37–53), and ranged from 30 to 70 years. Colorectal surgery prior to the start of CCS had been performed in two patients (5%), who both underwent appendectomy because of appendicitis. Twelve (32%) patients had a personal history of cancer. Three patients had a history of colorectal adenoma(s) at ages 39, 61, and 63, which were all non-advanced (Figure 2).

### 3.2. Detection and Yield of CCS

In total, 61 surveillance colonoscopies were performed in 37 patients, after excluding 6 incomplete colonoscopies that yielded no adenomas or CRC, which consisted of 37 baseline and 24 follow-up colonoscopies (Figure 1). The median age at first and last colonoscopy was 45 years (IQR: 41–54) and 47 years (IQR: 43–55), respectively (Table 1). Seventeen (46%) patients underwent multiple colonoscopies and had a median CCS follow-up time of 3 years (IQR: 2–5; Figure 2). No follow-up data were available for the remaining 20 patients as these patients were still awaiting a follow-up colonoscopy (17/20; 85%), passed away (2/20; 10%) or opted for CCS at another hospital closer to home (1/20; 5%). The total follow-up time was 67 years.

Of all 24 follow-up colonoscopies, 3 (13%) colonoscopies were performed with a 5-year interval, whereas 21 (87%) colonoscopies were performed with an altered interval (Table 2; Figure 2). For 19 out of 21 colonoscopies the interval was shortened, most often based on the gastroenterologist’s advice (i.e., findings of the previous colonoscopy or a combination of previous findings and colorectal complaints). For 2 out of 21 colonoscopies the interval was lengthened due to patient’s choice (i.e., priority for non-CRC related health problems or no show). The median CCS interval was 2 years (IQR: 1–4) and ranged between 3 months and 6 years. Of all 24 follow-up colonoscopies, 10 were performed with a 0–2-year interval when considering a margin of 1 month, and 14 with a 2–6 year interval.

During a total of 54 colonoscopies, 35 of 37 (95%) patients presented with at least one colorectal lesion (including adenomas, hamartomas NST, ganglioneuromas, juvenile polyps, lymphoid polyps, inflammatory polyps, hyperplastic polyps, and sessile serrated lesions; Table 3). More than 10 colorectal lesions per colonoscopy were found during 20 (33%) colonoscopies, and colorectal lesions were left in situ during 37 (61%) colonoscopies. Pathology-confirmed colorectal lesions were found in 33 (89%) patients during 48 colonoscopies, corresponding to a detection rate of 79 (95% CI: 66–88). Of these 33 patients, 17 (52%) presented with more than one type of colorectal lesion, and 7 (21%) with more than two types.

#### 3.2.1. Colorectal Carcinomas and Adenomas

**Figure 3:**
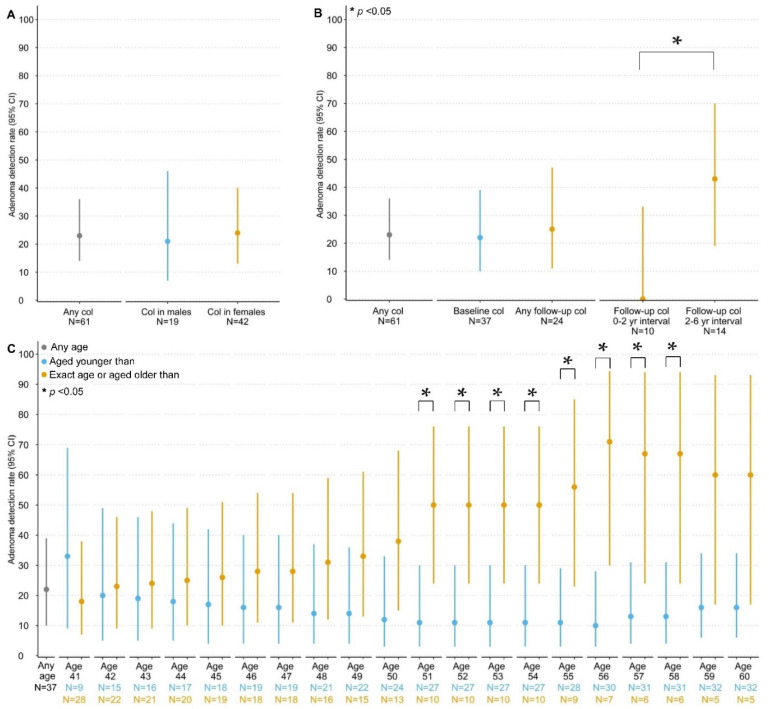
Differences in adenoma detection rates between (**A**) males and females, (**B**) baseline and follow-up, and (**C**) different age groups at baseline. Abbreviations: ADR: Adenoma detection rate; col: colonoscopies; CI: confidence interval. Adenoma detection rates represent the number of colonoscopies during which at least one adenoma was detected per 100 colonoscopies. (**C**): the relatively similar ADRs in the “aged younger than” group might be explained by a coincide of small sample size and small increase in the number of observed adenomas.

No (interval) CRCs were observed during CCS. In 13 (35%) patients a total of 29 adenomas were detected during 14 (23%) colonoscopies (Table 3; Figure 2). The median age at detection was 52 years (IQR: 43–60). Most adenomas were located in the proximal colon (11/14 colonoscopies; 79%). The corresponding overall adenoma detection rate (ADR) was 23 (95% CI: 14–36) per 100 colonoscopies. ADRs were similar between males and females and between baseline and follow-up (Figure 3). The ADR for follow-up colonoscopies performed within a 2–6 year interval (43, 95% CI: 19–70) was significantly higher than for follow-up colonoscopies performed within a 0–2 year interval (0, 95% CI: 0–34, *p* = 0.024). The ADRs did not differ by age from age 40 up until age 50, whereas colonoscopies performed at age ≥ 51 (40, 95% CI: 20–64) showed significantly higher ADRs compared with age < 51 (15, 95% CI: 6–30; *p* = 0.049). Baseline colonoscopies showed significantly higher ADRs when performed at age ≥ 51 (50, 95% CI: 24–76) compared with age < 51 (11, 95% CI: 3–30; *p* = 0.021; Figure 3). In contrast, the ADRs for follow-up colonoscopies did not differ by age.

Of all 13 patients, 1 presented with an advanced adenoma: a tubulovillous adenoma of 8 mm with low-grade dysplasia. The remainder of patients showed tubular adenomas of <10 mm with low-grade dysplasia. The median number of adenomas observed during a single colonoscopy was 1 (IQR: 1–2) and the median time to adenoma detection, which could be assessed in 6 patients, was 3 years (IQR: 3–5). The median size of the adenomas was 4 mm (IQR: 3–6), and ranged from 2 mm to 8 mm. Of all 13 patients, one had already been diagnosed with non-advanced adenomas prior to the start of CCS (Figure 2).

#### 3.2.2. Other Colorectal Lesions

Besides adenomas, hamartomas were found most often, in particular hamartomas NST and ganglioneuromas (Table 3). Hamartomas NST were detected during 18 colonoscopies in 16 (43%) patients at a median age of 51 years (IQR: 44–59). Ganglioneuromas were detected during 20 colonoscopies in 15 (41%) patients at a median age of 44 (IQR: 41–52). Detection rates for hamartomas NST and ganglioneuromas were 30 (95% CI: 19–43) and 33 (95% CI: 22–46) per 100 colonoscopies, respectively. Hamartomas NST were found in both the proximal and distal colon (9/17 colonoscopies; 53%) and ganglioneuromas mostly in the proximal colon (10/20 colonoscopies; 50%). Hyperplastic polyps and sessile serrated lesions of <10 mm without dysplasia were both detected in 7 (19%) patients (Table 3). Corresponding detection rates were 13 (95% CI: 6–25), and 13 (95% CI: 6–25) per 100 colonoscopies, respectively.

Male patients, who were slightly older at the last colonoscopy, had significantly higher detection rates of hamartomas NST (53, 95% CI: 29–75) compared with female patients (19, 95% CI: 9–35; *p* = 0.016). In addition, male patients had higher detection rates of ganglioneuromas (42, 95% CI: 21–66) compared with female patients (29, 95% CI: 16–45). No statistically significant or clinically relevant differences between females and males were found for any of the other colorectal lesions (Table S1).

## 4. Discussions

In this study, the detection and yield of CCS was evaluated in a cohort of 37 PHTS patients aged ≥ 40 without a history of CRC, of whom 17 (46%) patients underwent multiple colonoscopies and had a median CCS follow-up time of 3 years. CCS yielded no CRCs and only one advanced adenoma. Common colonoscopy findings were non-advanced adenomas, hamartomas NST, and ganglioneuromas, each detected in about 40% of the cohort. The adenoma yield at baseline was similar to follow-up and higher in patients aged older than 50 years.

The low CRC yield is likely due to the relatively young age of our cohort, especially given the previously reported median ages at CRC diagnosis in PHTS patients of 46 to 58 years [2], and might also be expected as current CRC risks are likely overestimated and as low as 0–5% up to age 50 [2]. Additionally, Khare et al. showed a low CRC yield during surveillance, with only 1 CRC at age 69 in a cohort of 65 patients with a mean age of 50 years. Nonetheless, these authors propose to start CCS at age 35–40, in line with the NCCN guideline [14], given that this is approximately 10 years before CRCs are observed in PHTS patients [6]. While CCS yielded no CRCs, it should be noted that three patients from our centre had a history of CRC prior to the PHTS diagnosis at ages 41, 43, and 71, which is in line with previous studies reporting PHTS patients who developed CRC before or around age 40 [25,26,27,28,29]. The low CRC yield might also be explained by the removal of adenomas during CCS, which might have contributed to CRC prevention as also shown in multiple previous studies [11,30,31,32]. Adenomas were diagnosed in 35% of our patients, which is in line with previous studies on PHTS patients (24–42%) [5,6,25]. The lower rate of 31% observed in a Dutch population-based cohort (median age of approximately 60 years) and the increasing prevalence of adenomas with age indicate that PHTS patients are more prone to develop adenomas [33,34]. Furthermore, some of our patients showed adenomas of 8 mm already at age 40, which were close to being classified as advanced, or showed adenomas before age 40. To ensure early detection and treatment of CRC and precursor lesions, which may benefit the prognosis and survival of patients [35,36,37], we propose to continue offering CCS to PHTS patients from age 40 onwards.

We observed differences in the occurrence of adenomas across patients at baseline and when considering that baseline colonoscopy findings are indicators of a patient’s future risk of CRC or advanced adenoma [38,39], we advise variable intervals. Based on literature findings in non-PHTS populations, individuals who present with an advanced or high number of adenomas or CRC are at increased risk of developing advanced neoplasia in the future compared with individuals without adenomas [38,39]. Variable intervals based on colonoscopy findings will likely contribute to optimised efficiency of CCS. Currently, variable intervals are also being applied within our centre and have been proposed by various PHTS CCS surveillance guidelines, though clear directions for the variable intervals are not yet provided [14,15,16,17]. For individuals without hereditary CRC syndromes guidelines on variable intervals exist and range between 1 and 10 years, depending on colonoscopy findings [23,40,41].

When translating this best available evidence to the PHTS population, we advise a 10-year interval for PHTS patients without adenomas or with 1–4 adenomas of <10 mm distal to the splenic flexure (i.e., low risk), and a 5-year interval for those with 1–4 adenomas of <10 mm including at least one adenoma proximal to the splenic flexure (i.e., intermediate risk) [23,40]. A 3-year interval is advised for patients presenting with ≥5 adenomas or ≥1 adenoma of ≥10 mm or high-grade dysplasia regardless of adenoma location (i.e., high risk), and an 1-year interval for those with CRC, after adequate treatment of these (pre)malignant lesions [23,40,41]. Besides adenomas, hamartomas NST and ganglioneuromas were found in 43% and 41% of our cohort, respectively, which demonstrates that hamartomas constitute the predominant type of colorectal lesions found in PHTS patients. These lesions, unlike adenomas, possibly harbour no malignant potential and hence justify a 10-year interval in the absence of intermediate or high risk adenomas. Yet when considering the high proportion of patients in whom not all lesions could be cleared (59%) and that differentiating between types of lesions during colonoscopy can be challenging, gastroenterologists might decide to shorten the recommended surveillance interval. This makes CCS complex and time consuming, and therefore, it is advisable to offer CCS in expertise centres or in close contact with PHTS expert gastroenterologists and pathologists.

Simulation of CCS in our cohort indicated a yield of about 0–0.5 patients with CRC and 1–2 patients with advanced adenoma at first follow-up, based on assumptions incorporating the intervals indicated above, and the incidence of CRC and advanced adenoma in relation to baseline findings [38]. In addition, about 90% of all patients would have been offered a longer interval compared with current practice. This simulation illustrates a significant reduction in colonoscopy burden (e.g., risk of complications, pain, and discomfort) while maintaining similar surveillance yield. Data from the general population suggest that most adenomas will never progress towards CRC and that the adenoma-to-carcinoma progression takes 10–15 years [42]. However, these data are not available for the PHTS population, and data from *PTEN* deficient mice suggest potentially faster tumour progression rates in PHTS patients [43]. Combining all available evidence, the proposed intervals might be considered justified in PHTS patients and can provide guidance for gastroenterologists, though careful evaluation of these intervals over time is highly advisable. Possible suspension of CCS might be considered after two consecutive colonoscopies without adenoma detection in patients with no history of (high-risk) adenomas. In contrast, within patients with a history of high risk adenomas or CRC, suspension might be considered at a certain age, for example age 75 [23].

This study is one of the first to report on the detection and yield of CCS in adult PHTS patients with extensive information on colorectal findings. CCS was performed at our recognised PHTS expertise centre where a considerable number of PHTS patients, acknowledging the rarity of PHTS, are being seen by our multidisciplinary team including PHTS expert gastroenterologists and pathologists. Nevertheless, our findings and subsequent recommendations should be interpreted with some caution, as the cohort was relatively young and the sample size and follow-up time were limited. About one-third of the cohort started CCS at age 50 or thereafter due to late PHTS diagnosis, which partly explains the limited follow-up time. In addition, no CRCs and only one advanced adenoma were found, which suggests that surveillance with less intensive intervals might also suffice. The low number of cases also hampered in-depth evaluation of progression times and hence follow-up intervals though clearly support reducing the frequency of surveillance. As a result, the proposed interval recommendations are mainly based on existing guidelines for individuals without hereditary CRC syndromes. Additionally, the reported median time to detection of non-advanced adenomas should be interpreted with some caution as this estimate is dependent on adenoma clearance at prior colonoscopy and timing of subsequent colonoscopy. Lastly, central pathology revision was not performed. However, the chance of missed CRCs and high-grade dysplasia is probably marginal.

## 5. Conclusions

In conclusion, colorectal cancer surveillance in 37 adult PHTS patients aged 40 onwards yielded no CRCs and only one advanced adenoma. Most common benign lesions, each observed in about 40%, were non-advanced adenomas, hamartomas, and ganglioneuromas. The low CRC and advanced adenoma yield allow for a more personalised surveillance program for PHTS patients, with less intensive intervals for patients without adenomas or only low-risk adenomas. Combining our data with literature findings on CRC risk and progression, we suggest starting CCS at age 40 and applying variable follow-up intervals between 1 and 10 years, depending on the occurrence of CRC and adenomas at the previous colonoscopy.

## Figures and Tables

**Table 1:** Characteristics of adult PHTS patients who started colorectal cancer surveillance.

	Patients (N = 37)	%
**General**		
Female gender, n/N (%)	24/37	65%
Index patient, n/N (%)	15/37	41%
Age at PHTS diagnosis, median (IQR)	41 (37–53)	
Age at first colonoscopy, median (IQR)	45 (41–54)	
Females	44 (40–50)	
Males	49 (41–60)	
Age at last colonoscopy, median (IQR)	47 (43–55)	
Females	46 (42–54)	
Males	50 (44–60)	
CCS follow-up time ^1^ (years), median (IQR)	3 (2–5)	
Females	4 (3–5)	
Males	3 (2–3)	
Colorectal surgery prior to the start of CCS, n/N (%)	2/37	5%
**PHTS-related clinical signs**		
Macrocephaly, n/N (%)	23/34	68%
Multiple oral lesions ^2^, n/N (%)	26/26	100%
Lhermitte-Duclos disease, n/N (%)	4/26	15%
Multiple skin lesions ^3^, n/N (%)	25/27	93%
Arteriovenous malformations, n/N (%)	6/26	23%
Colorectal lesions ^4^, n/N (%)	35/37	95%
Cancer, n/N (%)	12/37 *	32%
Thyroid	2/37	5%
Breast	9/24	38%
Colorectal	0/37 **	0%
Endometrial	2/24	8%
Melanoma	2/37	5%
Kidney	0/37	0%

Abbreviations: PHTS: *PTEN* Hamartoma Tumour Syndrome; CCS: Colorectal cancer surveillance; IQR: Interquartile range. ^1^ Assessed in patients with multiple colonoscopies. ^2^ Two out of the following oral lesions: gingival hypertrophy, high palate, and/or oral papillomas. ^3^ Two out of the following skin lesions: trichilemmoma, lipoma, fibroma, acanthosis nigricans, callus, corn, and/or pits. ^4^ Including any of the following lesions: adenomas, hamartomas of no special type, ganglioneuromas, juvenile polyps, lymphoid polyps, inflammatory polyps, hyperplastic polyps, and sessile serrated lesions. * Two patients presented with two types of cancer. ** Patients with a history of CRC were excluded.

**Table 2:** Alterations of the colorectal cancer surveillance interval in adult PHTS patients with follow-up colonoscopies.

	Patients with Follow-Up Colonoscopies (N = 17)	Follow-Up Colonoscopies (N = 24)
Surveillance with 5-year interval, n/N (%)		
No, shortened interval	14 (82%)	19 (79%)
No, lengthened interval	2 (12%)	2 (8%)
Yes	1 (6%)	3 (13%)
Reasons for shortened interval, n/N (%)		
Gastroenterologist’s advice ^1^	12 (86%)	15 (79%)
Patient’s choice ^2^	2 (14%)	4 (21%)
Reasons for lengthened interval, n/N (%)		
Gastroenterologist’s advice ^1^	0 (0%)	0 (0%)
Patient’s choice ^2^	2 (100%)	2 (100%)

Abbreviations: PHTS: *PTEN* Hamartoma Tumour Syndrome. ^1^ Findings of the previous colonoscopy or a combination of previous findings and colorectal complaints. ^2^ Colorectal complaints, priority for non-CRC related health problems, or no show.

**Table 3:** Detection and yield of colorectal cancer surveillance in adult PHTS patients.

	Patients (N = 37) ^3^	Colonoscopies (N = 61)
**Findings at colonoscopy**		
Colorectal lesion(s) * present	35 (95%)	54 (89%)
Number of lesions * present		
1–10	21 (57%)	25 (41%)
1–20	3 (8%)	6 (10%)
21–50	7 (19%)	10 (16%)
>50	3 (8%)	4 (7%)
Multiple unspecified	1 (3%)	9 (15%)
Lesions * left in situ	22 (59%)	37 (61%)
**Findings at pathology revision**		
Any colorectal lesion *		
Presence, N (%)	33 (89%)	48 (79%)
Detection rate (95% CI) ^1^		79 (66–88)
Colorectal carcinomas		
Presence, N (%)	0 (0%)	0 (0%)
Detection rate (95% CI) ^1^		0 (0–7)
Adenomas		
Presence, N (%)	13 (35%)	14 (23%)
Detection rate (95% CI) ^1^		23 (14–36)
Age at first detection, median (IQR)	52 (43–60)	
Location, N (%)	n.a.	
Proximal		11 (79%)
Distal		2 (14%)
Both proximal and distal		1 (7%)
Advanced adenoma, N (%) ^2^		1 (7%)
Number of adenomas, median (IQR)		1 (1–2)
Time to detection (years), median (IQR)		3 (3–5)
Hamartomas		
Hamartomas of no special type		
Presence, N (%)	16 (43%)	18 (30%)
Detection rate (95% CI) ^1^		30 (19–43)
Age at first detection, median (IQR)	51 (44–59)	
Location, N (%)	n.a.	
Proximal		2 (12%)
Distal		6 (35%)
Both proximal and distal		9 (53%)
Ganglioneuromas		
Presence, N (%)	15 (41%)	20 (33%)
Detection rate (95% CI) ^1^		33 (22–46)
Age at first detection, median (IQR)	44 (41–52)	
Location, N (%)	n.a.	
Proximal		10 (50%)
Distal		6 (30%)
Both proximal and distal		4 (20%)
Juvenile polyps		
Presence, N (%)	1 (3%)	1 (2%)
Detection rate (95% CI) ^1^		2 (0–10)
Age at first detection	40	
Location, N (%)	n.a.	
Proximal		0 (0%)
Distal		1 (100%)
Both proximal and distal		0 (0%)
Lymphoid polyps		
Presence, N (%)	3 (8%)	3 (5%)
Detection rate (95% CI) ^1^		5 (1–15)
Age at first detection, median (IQR)	47 (46–48)	
Location, N (%)	n.a.	
Proximal		1 (33%)
Distal		1 (33%)
Both proximal and distal		1 (33%)
Inflammatory polyps		
Presence, N (%)	7 (19%)	7 (11%)
Detection rate (95% CI) ^1^		11 (5–23)
Age at first detection, median (IQR)	42 (42–52)	
Location, N (%)	n.a.	
Proximal		4 (57%)
Distal		2 (29%)
Both proximal and distal		1 (14%)
Hyperplastic polyps		
Presence, N (%)	7 (19%)	8 (13%)
Detection rate (95% CI) ^1^		13 (6–25)
Age at first detection, median (IQR)	49 (43–52)	
Location, N (%)	n.a.	
Proximal		2 (25%)
Distal		6 (75%)
Both proximal and distal		0 (0%)
Sessile serrated lesions		
Presence, N (%)	7 (19%)	8 (13%)
Detection rate (95% CI) ^1^		13 (6–25)
Age at first detection, median (IQR)	46 (42–48)	
Location, N (%)	n.a.	
Proximal		6 (75%)
Distal		1 (13%)
Both proximal and distal		1 (13%)

Abbreviations: PHTS: *PTEN* Hamartoma Tumour Syndrome; CI: confidence interval; IQR: interquartile range; n.a.: not assessed. * Including any of the following lesions: adenomas, hamartomas of no special type, ganglioneuromas, juvenile polyps, lymphoid polyps, inflammatory polyps, hyperplastic polyps, and sessile serrated lesions. ^1^ Detection rates represent the number of colonoscopies during which at least one lesion was detected per 100 colonoscopies. ^2^ Adenomas with at least one of the following features: a ≥10 mm size, a villous or tubulovillous histology, or high-grade dysplasia. ^3^ For patients with multiple colonoscopies, lesions were considered present if detected during at least one colonoscopy.

## Data Availability

The data presented in this study are available on request from the corresponding author. The data are not publicly available due to privacy restrictions.

## Supplementary Materials

The following supporting information can be downloaded at: https://www.mdpi.com/article/10.3390/cancers14164005/s1, Table S1: Detection and yield of colorectal cancer surveillance in adult PHTS patients, stratified for gender.

## References

[1] Nelen M.R., Kremer H., Konings I.B., Schoute F., van Essen A.J., Koch R., Woods C.G., Fryns J.P., Hamel B., Hoefsloot L.H. (1999). Novel PTEN mutations in patients with Cowden disease: Absence of clear genotype-phenotype correlations. Eur. J. Hum. Genet..

[2] Hendricks L.A.J., Hoogerbrugge N., Schuurs-Hoeijmakers J.H.M., Vos J.R. (2021). A review on age-related cancer risks in PTEN hamartoma tumor syndrome. Clin. Genet..

[3] Yehia L., Keel E., Eng C. (2020). The Clinical Spectrum of PTEN Mutations. Annu. Rev. Med..

[4] Pilarski R. (2019). PTEN Hamartoma Tumor Syndrome: A Clinical Overview. Cancers.

[5] Heald B., Mester J., Rybicki L., Orloff M.S., Burke C.A., Eng C. (2010). Frequent gastrointestinal polyps and colorectal adenocarcinomas in a prospective series of PTEN mutation carriers. Gastroenterology.

[6] Khare A., Burke C.A., Heald B., O’Malley M., LaGuardia L., Milicia S., Cruise M., Eng C., Mankaney G. (2021). Endoscopic Findings in Patients With PTEN Hamartoma Tumor Syndrome Undergoing Surveillance. J. Clin. Gastroenterol..

[7] Stanich P.P., Pilarski R., Rock J., Frankel W.L., El-Dika S., Meyer M.M. (2014). Colonic manifestations of PTEN hamartoma tumor syndrome: Case series and systematic review. World J. Gastroenterol..

[8] The Dutch Population Cancer Screening Program Explanation for Registration of Colonoscopy Reports in the Context of the National Screening Program for Colorectal Cancer, Version 7.0, January 2022. https://www.bevolkingsonderzoeknederland.nl/media/1717/toelichting-registratie-coloscopieverslagen-70.pdf.

[9] Brenner H., Hoffmeister M., Stegmaier C., Brenner G., Altenhofen L., Haug U. (2007). Risk of progression of advanced adenomas to colorectal cancer by age and sex: Estimates based on 840,149 screening colonoscopies. Gut.

[10] Pinsky P.F., Schoen R.E. (2020). Contribution of Surveillance Colonoscopy to Colorectal Cancer Prevention. Clin. Gastroenterol. Hepatol..

[11] Zauber A.G., Winawer S.J., O’Brien M.J., Lansdorp-Vogelaar I., van Ballegooijen M., Hankey B.F., Shi W., Bond J.H., Schapiro M., Panish J.F. (2012). Colonoscopic Polypectomy and Long-Term Prevention of Colorectal-Cancer Deaths. N. Engl. J. Med..

[12] Shussman N., Wexner S.D. (2014). Colorectal polyps and polyposis syndromes. Gastroenterol. Rep..

[13] Dutch society of Clinical Genetics (VKGN) (2015). National Guideline PTEN Hamartoma Tumour Syndrome. Version 1.0. https://richtlijnendatabase.nl/gerelateerde_documenten/f/22306/IKNL%20richtlijn%20PTEN%20Hamartoom%20Tumor%20Syndroom.pdf.

[14] National Comprehensive Cancer Network Genetic/Familial High-Risk Assessment: Breast, Ovarian, and Pancreatic (Version 2.2022). https://www.nccn.org/professionals/physician_gls/pdf/genetics_bop.pdf.

[15] Boland C.R., Idos G.E., Durno C., Giardiello F.M., Anderson J.C., Burke C.A., Dominitz J.A., Gross S., Gupta S., Jacobson B.C. (2022). Diagnosis and Management of Cancer Risk in the Gastrointestinal Hamartomatous Polyposis Syndromes: Recommendations from the US Multi-Society Task Force on Colorectal Cancer. Gastroenterology.

[16] Tischkowitz M., Colas C., Pouwels S., Hoogerbrugge N., Bisseling T., Bubien V., Caux F., Chabbert-Buffet N., Colas C., Da Mota Gomes S. (2020). Cancer Surveillance Guideline for individuals with PTEN hamartoma tumour syndrome. Eur. J. Hum. Genet..

[17] UK Cancer Genetic Group UK Guidelines for Management for Tumour Risk in PTEN Hamartoma Syndrome. Version 1.0 May 2017. https://www.ukcgg.org/media/10879/pten_management_-_cgg_4may2017.pdf.

[18] Vos J.R., Hsu L., Brohet R.M., Mourits M.J., de Vries J., Malone K.E., Oosterwijk J.C., de Bock G.H. (2015). Bias Correction Methods Explain Much of the Variation Seen in Breast Cancer Risks of BRCA1/2 Mutation Carriers. J. Clin. Oncol..

[19] Raj K.P., Taylor T.H., Wray C., Stamos M.J., Zell J.A. (2011). Risk of second primary colorectal cancer among colorectal cancer cases: A population-based analysis. J. Carcinog..

[20] Phipps A.I., Chan A.T., Ogino S. (2013). Anatomic subsite of primary colorectal cancer and subsequent risk and distribution of second cancers. Cancer.

[21] Wallis Y., Payne S., McAnulty C., Bodmer D., Sistermans E., Robertson K., Moore D., Abbs S., Deans Z., Devereau A. (2013). Practice Guidelines for the Evaluation of Pathogenicity and the Reporting of Sequence Variants in Clinical Molecular Genetics. Association for Clinical Genetic Science and the Dutch Society of Clinical Genetic Laboratory Specialists. https://www.acgs.uk.com/media/10791/evaluation_and_reporting_of_sequence_variants_bpgs_june_2013_-_finalpdf.pdf.

[22] Protocol for Admission and Auditing of Colonoscopy Centers and Colonoscopists. National Bowel Cancer Screening Program. 2020 (Version 9.0). https://www.bevolkingsonderzoeknederland.nl/media/1493/20201207-protocol-toelating-en-auditing-cc-90.pdf.

[23] Dutch Society of Gastroenterologists and Pathologists (2013). Dutch Guideline Colonoscopy Surveillance. https://www.mdl.nl/files/richlijnen/Richtlijn_Coloscopie_Surveillance_definitief_2013.pdf.

[24] RStudio Team (2016). RStudio: Integrated Development for R.

[25] Bubien V., Bonnet F., Brouste V., Hoppe S., Barouk-Simonet E., David A., Edery P., Bottani A., Layet V., Caron O. (2013). High cumulative risks of cancer in patients with PTEN hamartoma tumour syndrome. J. Med. Genet..

[26] Tan M.-H., Mester J.L., Ngeow J., Rybicki L.A., Orloff M.S., Eng C. (2012). Lifetime cancer risks in individuals with germline PTEN mutations. Clin. Cancer Res..

[27] Riegert-Johnson D.L., Gleeson F.C., Roberts M., Tholen K., Youngborg L., Bullock M., Boardman L.A. (2010). Cancer and Lhermitte-Duclos disease are common in Cowden syndrome patients. Hered. Cancer Clin. Pract..

[28] Shaco-Levy R., Jasperson K.W., Martin K., Samadder N.J., Burt R.W., Ying J., Bronner M.P. (2017). Gastrointestinal Polyposis in Cowden Syndrome. J. Clin. Gastroenterol..

[29] Levi Z., Baris H.N., Kedar I., Niv Y., Geller A., Gal E., Gingold R., Morgenstern S., Baruch Y., Leach B.H. (2011). Upper and Lower Gastrointestinal Findings in PTEN Mutation-Positive Cowden Syndrome Patients Participating in an Active Surveillance Program. Clin. Transl. Gastroenterol..

[30] Winawer S.J., Zauber A.G., Ho M.N., O’Brien M.J., Gottlieb L.S., Sternberg S.S., Waye J.D., Schapiro M., Bond J.H., Panish J.F. (1993). Prevention of colorectal cancer by colonoscopic polypectomy. The National Polyp Study Workgroup. N. Engl. J. Med..

[31] Zhang J., Cheng Z., Ma Y., He C., Lu Y., Zhao Y., Chang X., Zhang Y., Bai Y., Cheng N. (2017). Effectiveness of Screening Modalities in Colorectal Cancer: A Network Meta-Analysis. Clin. Colorectal Cancer.

[32] Barrow P., Khan M., Lalloo F., Evans D.G., Hill J. (2013). Systematic review of the impact of registration and screening on colorectal cancer incidence and mortality in familial adenomatous polyposis and Lynch syndrome. Br. J. Surg..

[33] Bretthauer M., Kaminski M.F., Løberg M., Zauber A.G., Regula J., Kuipers E.J., Hernán M.A., McFadden E., Sunde A., Kalager M. (2016). Population-Based Colonoscopy Screening for Colorectal Cancer: A Randomized Clinical Trial. JAMA Intern. Med..

[34] Diamond S.J., Enestvedt B.K., Jiang Z., Holub J.L., Gupta M., Lieberman D.A., Eisen G.M. (2011). Adenoma detection rate increases with each decade of life after 50 years of age. Gastrointest. Endosc..

[35] Mehrkhani F., Nasiri S., Donboli K., Meysamie A., Hedayat A. (2009). Prognostic factors in survival of colorectal cancer patients after surgery. Colorectal Dis..

[36] Brenner H., Jansen L., Ulrich A., Chang-Claude J., Hoffmeister M. (2016). Survival of patients with symptom- and screening-detected colorectal cancer. Oncotarget.

[37] Friedrich K., Grüter L., Gotthardt D., Eisenbach C., Stremmel W., Scholl S.G., Rex D.K., Sieg A. (2015). Survival in patients with colorectal cancer diagnosed by screening colonoscopy. Gastrointest. Endosc..

[38] Lieberman D., Sullivan B.A., Hauser E.R., Qin X., Musselwhite L.W., O’Leary M.C., Redding T.S.T., Madison A.N., Bullard A.J., Thomas R. (2020). Baseline Colonoscopy Findings Associated With 10-Year Outcomes in a Screening Cohort Undergoing Colonoscopy Surveillance. Gastroenterology.

[39] Lee J.K., Jensen C.D., Levin T.R., Doubeni C.A., Zauber A.G., Chubak J., Kamineni A.S., Schottinger J.E., Ghai N.R., Udaltsova N. (2020). Long-term Risk of Colorectal Cancer and Related Death After Adenoma Removal in a Large, Community-based Population. Gastroenterology.

[40] Hassan C., Antonelli G., Dumonceau J.M., Regula J., Bretthauer M., Chaussade S., Dekker E., Ferlitsch M., Gimeno-Garcia A., Jover R. (2020). Post-polypectomy colonoscopy surveillance: European Society of Gastrointestinal Endoscopy (ESGE) Guideline–Update 2020. Endoscopy.

[41] Hassan C., Wysocki P.T., Fuccio L., Seufferlein T., Dinis-Ribeiro M., Brandão C., Regula J., Frazzoni L., Pellise M., Alfieri S. (2019). Endoscopic surveillance after surgical or endoscopic resection for colorectal cancer: European Society of Gastrointestinal Endoscopy (ESGE) and European Society of Digestive Oncology (ESDO) Guideline. Endoscopy.

[42] Jover R., Bretthauer M., Dekker E., Holme Ø., Kaminski M.F., Løberg M., Zauber A.G., Hernán M.A., Lansdorp-Vogelaar I., Sunde A. (2016). Rationale and design of the European Polyp Surveillance (EPoS) trials. Endoscopy.

[43] Marsh V., Winton D.J., Williams G.T., Dubois N., Trumpp A., Sansom O.J., Clarke A.R. (2008). Epithelial Pten is dispensable for intestinal homeostasis but suppresses adenoma development and progression after Apc mutation. Nat. Genet..

