# Supplementary material for: Detection and Yield of Colorectal Cancer Surveillance in Adults with PTEN Hamartoma Tumour Syndrome
Source: Cancers (Basel). 2022 Aug 19;14(16):4005. doi: 10.3390/cancers14164005 (PMC9406787; doi:10.3390/cancers14164005)
Supplement: Supplementary file 1 [file cancers-14-04005-s001.zip › cancers-1808114-supplementary.pdf]

cancers

MDPI

Supplementary Materials

Detection and Yield of Colorectal Cancer Surveillance in Adults with *PTEN* Hamartoma Tumour Syndrome

Meggie M. C. M. Drissen, Janet R. Vos, Dorien T. J. van der Biessen-van Beek, Rachel S. van der Post, Iris D. Nagtegaal, Mariëtte C. A. van Kouwen, Tanya M. Bisseling and Nicoline Hoogerbrugge

Table S1. Detection and yield of colorectal cancer surveillance in adult PHTS patients, stratified for gender.

	FEMALES		MALES	
	Patients (N=24) [3]	Colonoscopies (N=42)	Patients (N=13) [3]	Colonoscopies (N=19)
**Colorectal carcinomas**				
Presence, N (%)	0 (0%)	0 (0%)	0 (0%)	0 (0%)
Detection rate (95% CI) [1]		0 (0-10)		0 (0-21)
**Adenomas**				
Presence, N (%)	9 (38%)	10 (24%)	4 (31%)	4 (21%)
Detection rate (95% CI)[1]		24 (13-40)		21 (7-46)
Age at first detection, median (IQR)	51 (43-58)		57 (49-63)	
Location, N (%)	n.a.		n.a.	
Proximal		7 (70%)		5 (100%)
Distal		2 (20%)		0 (0%)
Both proximal and distal		1 (10%)		0 (0%)
Advanced adenoma, N (%)[2]		0 (0%)		1 (25%)
Number of adenomas, median (IQR)		1 (1-2)		1 (1-1)
Time to detection (years), median (IQR)		3 (3-5)		3
**Hamartomas**				
**Hamartomas of no special type**				
Presence, N (%)	8 (33%)	8 (19%)	8 (62%)	10 (53%)
Detection rate (95% CI) [1]		19 (9-35)		53 (29-75)
Age at first detection, median (IQR)	51 (44-54)		55 (46-62)	
Location, N (%)	n.a.		n.a.	
Proximal		2 (25%)		0 (0%)
Distal		3 (38%)		3 (33%)
Both proximal and distal		3 (38%)		6 (67%)
**Ganglioneuromas**				
Presence, N (%)	9 (38%)	12 (29%)	6 (46%)	8 (42%)
Detection rate (95% CI) [1]		29 (16-45)		42 (21-66)
Age at first detection, median (IQR)	41 (40-47)		48 (45-57)	
Location, N (%)	n.a.		n.a.	
Proximal		7 (58%)		3 (38%)
Distal		3 (25%)		3 (38%)
Both proximal and distal		2 (17%)		2 (25%)
**Juvenile polyps**				
Presence, N (%)	1 (4%)	1 (2%)	0 (0%)	0 (0%)
Detection rate (95% CI) [1]		2 (0-14)		0 (0-21)
Age at first detection	40		n.a.	
Location, N (%)	n.a.		n.a.	
Proximal		0 (0%)		n.a.
Distal		1 (100%)		n.a.
Both proximal and distal		0 (0%)		n.a.
**Lymphoid polyps**				
Presence, N (%)	1 (4%)	1 (2%)	2 (15%)	2 (11%)
Detection rate (95% CI) [1]		2 (0-14)		11 (2-35)
Age at first detection, median (IQR)	47		46 (44-47)	
Location, N (%)	n.a.		n.a.	
Proximal		1 (100%)		0 (0%)
Distal		0 (0%)		1 (50%)

Both proximal and distal		0 (0%)		1 (50%)
**Inflammatory polyps**				
Presence, N (%)	5 (21%)	5 (12%)	2 (15%)	2 (11%)
Detection rate (95% CI) [1]		12 (4-26)		11 (2-35)
Age at first detection, median (IQR)	45 (42-58)		42 (41-42)	
Location, N (%)	n.a.		n.a.	
Proximal		4 (80%)		0 (0%)
Distal		0 (0%)		2 (100%)
Both proximal and distal		1 (20%)		0 (0%)
**Hyperplastic polyps**				
Presence, N (%)	6 (25%)	7 (17%)	1 (8%)	1 (5%)
Detection rate (95% CI) [1]		17 (8-32)		5 (0-28)
Age at first detection, median (IQR)	47 (42-54)		49	
Location, N (%)	n.a.		n.a.	
Proximal		2 (29%)		0 (0%)
Distal		5 (71%)		1 (100%)
Both proximal and distal		0 (0%)		0 (0%)
**Sessile serrated lesions**				
Presence, N (%)	4 (17%)	5 (12%)	3 (23%)	3 (16%)
Detection rate (95% CI) [1]		12 (4-26)		16 (4-40)
Age at first detection, median (IQR)	47 (45-54)		44 (42-46)	
Location, N (%)	n.a.		n.a.	
Proximal		4 (80%)		2 (67%)
Distal		1 (20%)		0 (0%)
Both proximal and distal		0 (0%)		1 (33%)

Abbreviations: PHTS: *PTEN* Hamartoma Tumour Syndrome; CI: confidence interval; IQR: interquartile range; n.a.: not assessed. [1] Detection rates represent the number of colonoscopies during which at least one lesion was detected per 100 colonoscopies. [2] Adenomas with at least one of the following features: a ≥10 mm size, a villous or tubulovillous histology, or high-grade dysplasia. [3] For patients with multiple colonoscopies, lesions were considered present if detected during at least one colonoscopy.
